# Allergic rhinitis improvement after septorhinoplasty in a sample of allergic rhinitis patients with septal deviation: a quasi-experimental study

**DOI:** 10.1590/1516-3180.2021.0236.R1.03052021

**Published:** 2021-11-29

**Authors:** Vanesa García-Paz, Cintia Micaela Chamorro-Petronacci, Roi Painceira-Villar, Ricardo Becerro-de-Bengoa-Vallejo, Marta Elena Losa-Iglesias, Mario Pérez-Sayáns, Adolfo Sarandeses-Garcia, Daniel López-López

**Affiliations:** I MD. Research, Health and Podiatry Group, Department of Health Sciences, Faculty of Nursing and Podiatry, Universidade da Coruña, A Coruña, Spain; Area Specialist Physician, Department of Allergology, Complejo Hospitalario Universitario de A Coruña (CHUAC), A Coruña, Spain.; II PhD, DDS. Researcher, Instituto de Investigaciones Sanitarias de Santiago (IDIS), Santiago de Compostela, Spain; Researcher Fellow, MedOralRes (Grupo de Investigación en Patología Oral Médico Quirúrgica), Universidad de Santiago de Compostela, A Coruña, Spain.; III DP, MSc, PhD. Research, Health and Podiatry Group, Department of Health Sciences, Faculty of Nursing and Podiatry, Universidade da Coruña, Ferrol, Spain.; IV RN, BSc, MLIS, DPM, DHL, PhD. Full Professor, Facultad de Enfermería, Fisioterapia y Podología, Universidad Complutense de Madrid, Madrid, Spain.; V RN, DHL, BSc, MSc, PhD, DPM. Full Professor, Faculty of Health Sciences, Universidad Rey Juan Carlos, Alcorcón, Spain.; VI PhD, DDS. Researcher, Instituto de Investigaciones Sanitarias de Santiago (IDIS), Santiago de Compostela, Spain; Research Fellow, MedOralRes (Grupo de Investigación en Patología Oral Médico Quirúrgica), Universidad de Santiago de Compostela, A Coruña, Spain.; VII PhD, MD. Professor, Otorhinolaryngology, Escuela Universitaria (EU), Universidade da Coruña, A Coruña, Spain. Supervision; VIII MSc, BSc, PhD, DPM. Senior Lecturer and Researcher, Health and Podiatry Group, Department of Health Sciences, Faculty of Nursing and Podiatry, Universidade da Coruña, Ferrol, Spain.

**Keywords:** Rhinitis, allergic, Quality of life, Rhinomanometry, Septal deviation, Health-related quality of life, Pathogenesis of rhinitis

## Abstract

**BACKGROUND:**

Allergic rhinitis (AR) is a chronic inflammatory disease that affects almost 30% of the adult population.

**OBJECTIVE:**

To describe and compare the evolution of symptoms in patients diagnosed with AR and septal deviation prior to and following septoplasty (STP).

**DESIGN AND SETTING:**

Quasi-experimental study developed in A Coruña University Hospital.

**METHODS:**

Patients aged 18-65 years who had been diagnosed with AR and septal deviation were recruited. Obstruction airflow was evaluated before and after surgery, by means of anterior rhinomanometry (RNM). Severity symptoms and quality of life were assessed using a visual analogue scale (VAS) and the ESPRINT questionnaire, respectively.

**RESULTS:**

A total of 50 subjects underwent STP and 42 were included in this study. Their mean age at the time of surgery was 34.16 ± 9.74 years (range 18-64). Significant reductions in mean VAS and ESPRINT were observed after surgery (P < 0.01). These outcomes were considered to represent an overall improvement in quality of life. The RNM results also improved significantly, from mean values of 478.07 ± 165.4 cm^3^/s before STP to 826.4 ± 175.5 cm^3^/s afterwards (P < 0.01).

**CONCLUSIONS:**

The negative correlations of VAS and ESPRINT with RNM, from before and to after STP, demonstrate the efficacy of scales and questionnaires as objective methods for determining obstruction in the absence of rhinomanometry. Patients with allergic rhinitis and septal deviation showed improvements in obstruction severity and medication use after STP.

## INTRODUCTION

Allergic rhinitis (AR) is a chronic inflammatory disease that affects almost 30% of the adult population and is associated with other inflammatory diseases.^1^ In AR, the upper airway respiratory mucosa becomes inflamed in response to allergen exposure, mediated by the T helper 2 (Th2) immunological response. Common symptoms include sneezing, rhinorrhea, nasal congestion, nasal itching and nasal obstruction. Nasal obstruction is the symptom that is most refractory to medical treatment.^2^

The evolution of AR includes chronic rhinosinusitis, nasal polyposis or chronic rhinitis.^3^ Despite the high prevalence of AR and the variety of medications for treating it, many patients still feel that their treatment has failed.^4^ AR impairs quality of life (QoL) and work productivity, since it is an important cause of absence from work and school, and it generates huge costs in prescription medication.^5^

Deviation of the nasal septum is diagnosed in more than 70% of the general population to some degree.^6^ It causes symptoms such as nasal obstruction, epistasis, snoring, anxiety, headaches, buccal breathing and sinusitis.^7,8^ Septorhinoplasty (STP) is the most common treatment for patients with septal deviation and generally gives rise to satisfactory outcomes.^9^

The treatments for AR include avoidance of the causative allergen, a great variety of medications (such as antihistamines, anti-leukotrienes or corticoids) and specific allergen immunotherapy. These conservative methods lead to improvement of symptoms but, for some refractory patients, medication alone is not enough. The surgical procedures for treating AR include cryotherapy, laser cautery, sinus surgery or turbinate resection. AR guides do not nowadays include STP as a therapeutic possibility, given that its outcomes are not as satisfactory as in patients who only show septal deviation.^10^

Kim et al. used two different AR groups to study how STP and turbinoplasty or turbinoplasty alone affected the evolution of their patients’ disease.^11^ They observed that the use of medication was diminished in both groups, with improvements in VAS (visual analogue scale (VAS) scores, although they did not measure airflow rates.

AR patients with septal deviation present a therapeutic challenge for physicians. The current guides for AR management underscore the need for research regarding the role of STP in the evolution of AR.^12^

## OBJECTIVE

Our main objective was to describe and compare the impact of AR symptoms prior to and following STP. It was hypothesized that STP would modify the clinical course of AR in patients presenting an association between this entity and septal deviation, and that STP would reduce the use of medication.

## METHODS

### Design and sample

This was a prospective quasi-experimental non-randomized controlled pre and post-test study. Informed consent was obtained from all patients, in accordance with the Declaration of Helsinki of 1975, as revised in 2013. This study was approved by the Regional Research Ethics Committee of Galicia (Ref: 2015/280; June 30, 2015).

The sample size was determined as follows. We estimated in relation to “severity” that before the intervention, 60% of the patients presented moderate-severe rhinitis and that this percentage would be expected to be reduced to 30% after 12 months. Thus, 51 patients would be required to detect this difference as statistically significant with a safety of 95% and a statistical power of 80%, in a bilateral approach with paired data. Regarding the variable of VAS, we assumed from pilot experience that the mean score prior to surgery was around 7.5 and that the decrease could be to 7 with a standard deviation of 1.87. Thus, 46 patients would be required to detect this difference as significant with a safety of 95%, a statistical power of 80%, in a bilateral approach with paired data.

Patients aged 18-65 years who presented a diagnosis of AR and septal deviation were recruited through consecutive sampling. They underwent STP at the Allergology and Otorhinolaryngology service of A Coruña University Hospital (CHUAC).

The diagnosis of septal deviation was determined from rhinofibroscopy findings. The diagnosis of AR was established clinically, with testing for specific allergen sensitization, e.g. correlations with symptoms and skin prick test positivity > 3 mm. The allergens tested were all common aeroallergens in our environment, such as dust mites, pollens from grasses, weeds and trees, fungi (such as *Aspergillus*, *Alternaria* and *Cladosporium*), cat and dog epithelia, latex and panallergens such as profilin and lipid transporter protein (LTP), through commercial extracts from ALK Abello Laboratories (Madrid, Spain).

The patients needed to have had AR symptoms for at least one year. Their symptoms were classified in accordance with the Allergic Rhinitis and its Impact on Asthma (ARIA) guidelines,^13^ i.e. “intermittent AR” (symptoms on less than four days per week) or “persistent AR” (symptoms on more than four days per week).

The severity of the patient’s symptoms was classified as “moderate-severe” if the patient reported having sleeping disorders, impairment of daily activity or absence from work or school, or “mild” if the patient had none of these.

Patients were excluded from the study in the following situations: less than one year of AR symptoms; already treated with specific immunotherapy against allergens; undergoing simultaneous surgical procedure (e.g. turbinate reduction); smokers; or presentation of chronic obstructive pulmonary disease, psychiatric disorders, malignant tumors, severe hepatopathy, obstructive sleep apnea or previous nasal surgery.^14,15^

### Procedure

Patient enrolment, surgical procedures and parameter evaluation were performed by the same surgeon (VGP). Variables were collected from the patients’ clinical history before and four weeks after surgery.

After screening, each patient’s obstruction was evaluated by means of anterior rhinomanometry (RNM) (cm^3^/s), using the Rhinospir Pro-165 device (Sibelmed, Barcelona, Spain). It was classified as mild, moderate, severe or very severe, according to the rhinomanometric grading.

QoL was scored through the ESPRINT scale. ESPRINT is a validated Spanish questionnaire on symptoms, activities of daily living, sleep disorders, psychology and overall health perception for AR patients.^16^

Symptoms like sneezing, itchy nose, ocular symptoms and nasal obstruction were evaluated using a visual analogue scale (VAS) and the clinical history. VAS scores were classified as mild (scores of 1-3), moderate (4-6) or severe (7-10).

Use of medications (intranasal corticosteroid, anti-leukotrienes, antihistamine eye drops or oral antihistamine) and their frequency of use were registered.

### Statistical analysis

All data were collected in a database and were analyzed using the Statistical Package for the Social Sciences (SPSS), version 20.0 (SPSS Inc., Chicago, Illinois, United States).

The standard deviation (SD) or interquartile range (IQR) were used to describe quantitative variables. For categorical variables, the frequency and percentage were used. Sample normality was assessed using the Kolmogorov-Smirnov test. Univariate analysis was used to compare measurements, with either Student‘s t distribution or the Mann-Whitney U test, depending on the application conditions. To compare proportions, the chi-square test or Fisher‘s exact test was used. A correlation analysis was carried out between quantitative variables using the Fisher or Spearman statistical test, according to the application conditions. Multiple regression models were applied, with percentage changes in airflow as the dependent variable, to assess whether these differed according to the severity of AR, with adjustments for age and sex. The significance level was set at P < 0.05.

## RESULTS

A total of 50 patients underwent STP and 42 of them agreed to participate in this study, comprising 27 males (64.3%) and 15 females (35.7%). Their mean age at the time of the surgery was 34.16 ± 9.74 years (range 18-64). Among the age groups, the largest group was the patients who were younger than 30 years (37.2%) (Table 1).

**Table 1.** Sociodemographic and clinical characteristics of the sample population

	Total group Mean ± SD Range n = 42	Male Mean ± SD Range n = 27	Female Mean ± SD Range n = 15	P-value Male versus female
**Age**	34.16 ± 9.7 (18-64)	31.63 ± 10.3 (18 -64)	38.73 ± 6.7 (29-50)	0.04
**VAS score (prior to STP)**	8.52 ± 1.1 (6-10)	8.56 ± 1 (7-10)	8.47 ± 1.3 (6-10)	0.8
**VAS score (after STP)**	3.74 ± 2.1 (1-8)	3.67 ± 2.2 (1-8)	3.87 ±1.8 (2-7)	0.77
**ESPRINT score (prior to STP)**	62.24 ± 14.84 (9 – 88)	60.89 ± 17.3 (9-88)	64.67 ± 8.93 (46-76)	0.43
**ESPRINT score (after STP)**	23.48 ± 18.8 (0-77)	21.93 ± 21.85 (0-77)	26.27 ± 11.74 (9-46)	0.48
**Flow rate (prior to STP)**	478.07 ± 165.4 (142-1242)	461.63 ± 123.7 (142-710)	507.67 ± 224.1 (287-1242)	0.39
**Flow rate (after STP)**	826.4 ± 175.5 (520-1340)	823.63 ± 178.2 (520-1265)	831.4 ± 176.46 (610-1340)	0.8

SD = standard deviation; VAS = visual analogue scale; STP = septoplasty.

All surgeries were conducted without complications and all patients were discharged in less than 24 hours. Follow-up was performed four weeks after surgery.

Allergies to beta lactamase (eleven patients) and non-steroidal anti-inflammatory drugs (NSAIDs) (two patients) allergic were the most common comorbidities (n = 13, 30.95%).

The allergen most recorded among the patients was dust mites (n = 33; 78.6%), followed by dust mites and pollen (n = 8; 19%) and pollen (n = 1; 2.4%).

The clinical data recorded prior to and following surgery are summarized in Table 2.

**Table 2.** Clinical variables before and after surgery

Variable	Before surgery	After Surgery	P-value
**Nasal obstruction**	n = 42 (100%)	n = 21 (50%)	< 0.01
**Sneezing**	n = 42 (100%)	n = 42 (100%)	–
**Rhinorrhea**	n = 41 (97.60%)	n = 28 (68.3%)	**< 0.01**
**Ocular symptoms**	n = 23 (54.8%)	n = 4 (9.5%)	**< 0.01**
**Nasal corticoid**	n = 41 (97.6%)	n = 18 (42.9%)	**< 0.01**
**Antihistamine**	n = 41 (97.6%)	n = 28 (66.7%)	**< 0.01**
**Anti-leukotriene**	n = 15 (35.7%)	n = 1 (2.4%)	**< 0.01**
**Intermittent AR**	n = 2 (4.8%)	n = 2 (4.8%)	0.12
**Persistent AR**	n = 40 (95.2%)	n = 12 (28.6%)	**< 0.01**
**Mean VAS score**	8.52 ± 1.13	3.74 ± 2.1	**0.02**
**Mean ESPRIT score**	62.24 ± 14.84	23.48 ± 18.28	**< 0.01**
**RNM (cm^3^/s)**	478.07 ± 165.4	826.4 ± 175.5	**< 0.01**
**RNM classification**			**0.037**
Normal	0	n = 14 (33%)	
Mild	n = 1 (2.4%)	n = 21 (50%)	
Moderate	n = 12 (28.6%)	n = 7 (16.7%)	
Severe	n = 18 (42.9)	0	
Very severe	n = 11 (23.8%)	0	
**ARIA classification of severity**			**0.04**
None	0	n = 34 (81%)	
Mild	n = 24 (57.1%)	n = 8 (19%)	
Moderate-severe	n = 18 (42.9%)	0	

AR = allergic rhinitis; VAS = visual analogue scale; RNM = anterior rhinomanometry; ARIA = allergic rhinitis and its impact on asthma. X-axis: patient code; y-axis: flow rate rhinomanometry, cm^3^/seconds.

**Figure 1.**
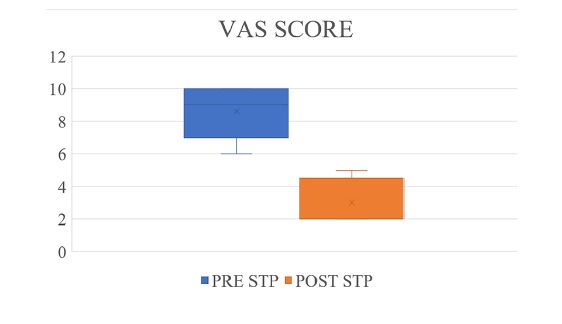
Evolution of visual analogue scale (VAS) results from before to after septoplasty (STP).

Regarding VAS scores before STP, most patients (97.6%) reported having severe symptoms (scores of 7-10) (Figure 1). After STP, most patients reported having mild symptoms (46.5%).

**Figure 2.**
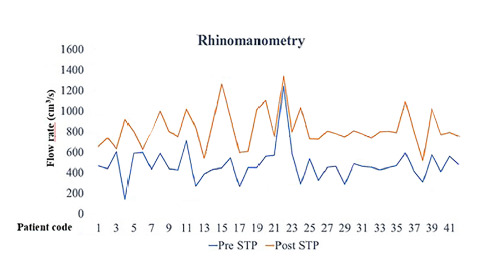
Evolution of anterior rhinomanometry on patients from before to after septoplasty (STP).

RNM measurements showed statistically different mean values among the operated patients, as can be seen in Figure 2 (RNM evolution). Airflow changes were found to be correlated with sex and age (P < 0.01 and P = 0.03) and with previous RNM severity (P = 0.01), i.e. patients whose severity of symptoms before the surgery was worse underwent greater percentage improvements.

X-axis: patient code; y-axis: flow rate rhinomanometry, cm^3^/seconds.

Among the operated women, 93% showed changes in temporality, from persistent to intermittent, while 59.2% of the men reported this evolution (P = 0.019).

Patients who were more than 40 years old reported worse outcomes regarding improvement of ocular symptoms (P = 0.049). Thus, 27% of these individuals continued to have these symptoms after surgery, while none of the patients aged between 30 and 40 years still had them.

Comorbidities, medication use, previous RNM severity or specific allergens were not associated with any improvement parameter (P > 0.05).

### Correlation analysis

The correlations demonstrated the effectiveness of both self-assessment methods (VAS and ESPRINT tools) for evaluating symptoms and QoL before STP (r = 0.32; P = 0.04) and after STP (r = 0.69; P < 0.01).

The negative correlations of VAS and ESPRINT with RNM before STP (r = -0.29, P = 0.05; and r = -0.31, P < 0.01) and after STP (r = -0.43, P < 0.01; and r = -0.43, P = 0.05) demonstrated the efficacy of the scales and questionnaires as objective methods for determining obstruction in the absence of rhinomanometry.

## DISCUSSION

A total of 42 patients diagnosed with AR and septal deviation were included in this study. We analyzed STP outcomes through objective measurements of nasal patency, QoL and symptoms, using questionnaires, and most patients (n = 32; 76.1%) reported obtaining improvements in all these fields.

According to recent studies, patients with AR will feel less benefit and satisfaction after STP.^10,17,18^ Physicians are usually faced with a dilemma when they have to decide how to manage nasal obstruction in AR patients, especially if this obstruction is incomplete. Two etiologies for the obstructive/congestive nasal symptoms of patients with allergic rhinitis and septal deviation are possible: one inflammatory and other structural. Accordingly, there should be distinct medical and surgical treatment considerations for each of these.^18^ For this reason, we decided to only include patients with more than one year of symptoms for whom medication treatment had already been established. Independently of the AR diagnosis, previous studies with different objective and subjective methods have demonstrated that a general improvement in nasal obstruction is achieved after STP.^8,9,11,18,19,20^

The results regarding determination of AR as a predictive factor for less improvement in STP have been contradictory in recent studies. Mondina et al. studied 100 patients after STP, among whom 28 presented AR, using the NOSE and RhinoQoL questionnaires before and after surgery. They concluded that AR was a predictive factor for less improvement,^21^ as was also demonstrated by Kartzanis et al.^10^ However, these outcomes are contradictory, considering that Stewart et al. could not prove this relationship.^22^

Confounding variables need to be considered cautiously. Improvements in AR symptoms could be due to use of topical antiallergic medication that can reach the nasal mucosa better after surgery. It is important to consider that there is some superposition of obstruction symptoms, given that they cannot be uniquely associated with any anatomical component or allergic component.^18^

Regarding sneezing, STP had no influence on sneezing frequency in our study, as none of our patients showed improvement in this symptom. Nevertheless, other authors have achieved significant changes in this symptom after STP.^9^ Improvements in other symptoms such as rhinorrhea or ocular symptoms (i.e. itching or watery eyes) were reported by our patients, in the same way as shown by Faulcon et al.^23^

Recent classifications have used the frequency of symptoms (number of days per week) to determine the type of AR.^24^ Most of our patients (95.1%) reported having persistent AR before surgery, and 71.4% of them improved to intermittent AR. It is important to underscore that complete resolution of AR was not registered in any patient, given that the original factor that generated AR, i.e. the allergen, was not eliminated.

Reductions in medication use (corticoids, antihistamines or anti-leukotrienes) were observed among the operated patients. Decreased need for corticoids was associated with improvement of obstruction symptoms (P < 0.01), although this relationship was not found with antihistamine or anti-leukotriene reduction. Improvement in rhinorrhea was associated with reduction of antihistamine use (P = 0.019). To the best of our knowledge, this was the first study to evaluate anti-leukotriene use among AR patients before and after STP.

AR impairs basic activities of daily life (BADL) in 81.8% of patients and affects their work capacity and social relationships.^25^ The prevalence of AR is increasing around the whole world and is generating economic and social impairments, including medical expenditure and diminished work productivity. AR treatment aims to reduce symptoms and improve QoL. Although STP does not eliminate central inflammatory AR, the additional permeability obtained reduces edematous mucosa and can relieve symptoms.

The VAS results highlighted the improvement that patients experienced after surgery. This visual scale has also been used in other studies. Kim et al. conducted a study similar to ours, in which better VAS results after STP were found among AR patients.^11^ ESPRINT is a validated questionnaire on quality of life, and its correlation with VAS indicates the relationship between patients’ symptoms and their BADL (r = 0.32; P = 0.043). Both ESPRINT and VAS showed negative correlations with the RNM results. Demoly et al. validated the VAS scale as presenting high sensitivity, in a study on 100 patients, using RNM.^26^ However, some other researchers such as Lara-Sánchez et al., in a prospective study on 102 patients, did not find any correlation with VAS and RNM.^27^

In some studies, the improvement obtained from before to after surgery was greater ten years after surgery (83%) than six months after it (69%).^28^ The length of follow-up has usually ranged from one month to ten years, although most authors agree that one month provides enough time to judge the surgical outcome.^10,29^ Since the level of patient satisfaction in our study was higher than 69%, our intention is to continue to make measurements annually over a five-year period.

The limitations of our study include its sample, given that the initial calculation for the sample size required for comparison of AR severity and VAS (assuming 95% certainty, 80% statistical power and 10% losses) indicated that sample required was 46 patients. Initially, 50 patients were included in this study but eight of them could not come to the post-test appointment, because of geographical and legal situations. Another possible limitation of this study was the lack of records regarding nasal decongestant use. Decongestants are medications that are used frequently through patients’ own decisions, without medical indication. Use of this medication induces vasoconstriction and rapid symptom relief, but has no effect on other symptoms.^30^

STP has traditionally been discarded as a surgical modality for AR patients, given that they do not achieve improvement of their breathing, unlike patients who only present septal deviation.^10^ However, some authors and current guidelines^11,12^ agree that work in this field is still required. From our results, we recommend that STP should be used for patients with AR and septal deviation, particularly for refractory patients.

## CONCLUSIONS

The negative correlation of VAS and ESPRINT with RNM from before to after STP demonstrates the efficacy of scales and questionnaires as objective methods for determining occurrences of obstruction in the absence of rhinomanometry. Patients with allergic rhinitis and septal deviation showed improvements in their severity of obstruction and reductions of their medication use after STP.
